# Oral Adelmidrol Administration Up-Regulates Palmitoylethanolamide Production in Mice Colon and Duodenum through a PPAR-γ Independent Action

**DOI:** 10.3390/metabo12050457

**Published:** 2022-05-19

**Authors:** Alessandro Del Re, Irene Palenca, Luisa Seguella, Marcella Pesce, Chiara Corpetti, Luca Steardo, Sara Rurgo, Giovanni Sarnelli, Giuseppe Esposito

**Affiliations:** 1 Department of Physiology and Pharmacology V. Erspamer, Sapienza University of Rome, Piazzale AldoMoro 5, 00185 Rome, Italy; alessandro.delre@uniroma1.it (A.D.R.); irene.palenca@uniroma1.it (I.P.); luisa.seguella@uniroma1.it (L.S.); chiara.corpetti@uniroma1.it (C.C.); 2 Department of Clinical Medicine and Surgery, University of Naples Federico II, 80131 Naples, Italy; mapesc@hotmail.com (M.P.); sararurgo91@gmail.com (S.R.); sarnelli@unina.it (G.S.); 3 Department of Psychiatry, Giustino Fortunato University, 82100 Benevento, Italy; luca.steardo@uniroma1.it

**Keywords:** adelmidrol, ALIAmides, palmitoylethanolamide, entourage effect, PPAR-γ

## Abstract

Adelmidrol is a promising palmitoylethanolamide (PEA) analog which displayed up-and-coming anti-inflammatory properties in several inflammatory conditions. Recent studies demonstrated that Adelmidrol is an in vitro enhancer of PEA endogenous production, through the so called “entourage” effect. The present study investigated the ability of Adelmidrol (1 and 10 mg/Kg per os) to increase the endogenous level of PEA in the duodenum and colon of mice after 21-day oral administration in the presence and absence of PPAR-γ inhibitor (1 mg/kg). The level of PEA was analyzed by HPLC-MS. The expression of PEA-related enzymatic machinery was evaluated by western blot and RT-PCR analysis. Our findings demonstrated that Adelmidrol significantly increased PEA levels in the duodenum and colon in a dose/time-dependent manner. We also revealed that Adelmidrol up regulated the enzymatic machinery responsible for PEA metabolism and catabolism. Interestingly, the use of the selective irreversible PPAR-γ antagonist did not affect either PEA intestinal levels or expression/transcription of PEA metabolic enzymes following Adelmidrol administration. The “entourage effect” with Adelmidrol as an enhancer of PEA was thus PPAR-γ-independent. The findings suggest that Adelmidrol can maximize a PEA therapeutic-based approach in several intestinal morbidities.

## 1. Introduction

**Figure 1:**
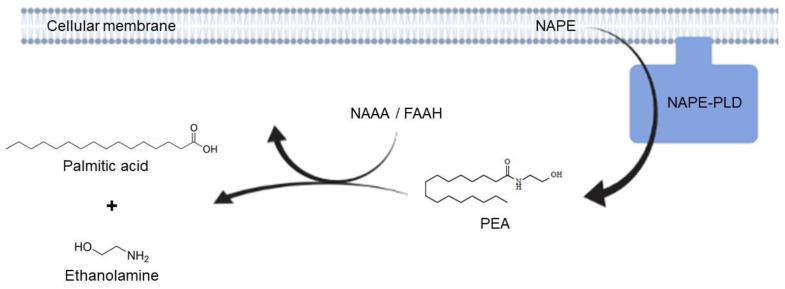
Schematic representation of PEA biosynthesis and catabolism. NAPE = N-acyl phosphatidylethanolamine; PLD = Phospholipase D; PEA = Palmitoylethanolamide; NAAA = N-acylethanolamine-hydrolyzing acid amidase; FAAH = Fatty acid amide hydrolase.

Palmitoylethanolamide (PEA) is the parent molecule of ALIAmides (Autacoid Local Injury Antagonist amides), i.e., lipid compounds belonging to the N-acylethanolamines (NAEs). PEA is produced on-demand by the enzyme N-acyl phosphatidylethanolamine phospholipase D (NAPE-PLD) in response to damage and is in situ degraded by the two catabolic enzymes N-acylethanolamine-hydrolyzing acid amidase (NAAA) and Fatty acid amide hydrolase (FAAH) (see Figure 1). PEA is involved in the regulation of several physiological processes, mostly due to the agonism on the peroxisome proliferator–activated receptor-α (PPAR-α) [1]. PEA exerts a wide range of anti-inflammatory effects, downregulating inducible nitric oxide synthase (iNOS), cycloxigenase-2 (COX-2), tumor necrosis factor-α (TNF-α) expression, NF-κB and Tool like receptor 4 (TLR4) signaling pathways, with downstream regulation of pro-inflammatory cytokines and immune cell infiltration in inflamed tissues. PEA anti-inflammatory effects have been tested in several in vitro and in vivo models of colitis, as well as gastrointestinal biopsies from human patients with inflammatory bowel disease (IBD) and functional dyspepsia [2,3,4,5]. Interestingly, the PEA safety and tolerability profile is very favorable in human and animal patients [6,7,8,9], especially if subjected to particle size reduction [10]. On the other hand, one of the main limitations to the use of PEA in clinical practice lies in its poor pharmacokinetic profile, due to marked lipophilicity that limits solubility and in vivo absorption. Although some new formulations of PEA (micronized and ultramicronized forms) seem to show more favorable absorption kinetics [11,12], new research efforts to overcome pharmacokinetic limitations have focused on molecules capable of triggering the so-called “entourage” effect, i.e., the ability of some ALIAmides and cannabinoids to increase the endogenous levels and/or activity of endocannabinoid compounds [13,14] by inhibiting their catabolic pathways, increasing the biosynthetic ones and/or enhancing their receptor affinity. In this framework, Adelmidrol is a promising ALIAmide and a semi-synthetic analog of PEA. It is the ethanolamide of the azelaic acid, a phytotherapic compound present in grain cereals and found to be topically effective in human inflammatory skin disorders [15], probably through the down-modulation of keratinocyte inflammatory responses [16]. Adelmidrol displayed up-and-coming pre-clinical effects in a variety of models of inflammation [17,18,19,20,21,22,23,24], that seem to depend, at least in part, upon PPAR-γ agonism [18]. Furthermore, a recent study demonstrated that Adelmidrol is an in vitro enhancer of PEA levels, probably thanks to an “entourage” effect [25]. It is possible to hypothesize that adelmidrol may act as a metabolic precursor of PEA or, more likely, it may exert an enhancing effect on the biosynthetic machinery of PEA. Finally, considering the perspective suggested by Cordaro et al. [22] on the possible use of Adelmidrol as a side-on-treatment in IBD, further information on the ability of such ALIAmide to actually change intestinal PEA levels would be fascinating.

The present study aimed to investigate whether orally administered Adelmidrol increases the levels of PEA in colonic and duodenal biopsies of mice and if PPAR-γ agonism is involved. A selective irreversible PPAR-γ antagonist, GW9662, was used to this end.

## 2. Results

### 2.1. Adelmidrol Increases Endogenous Levels of PEA in a Dose/Time-Dependent Manner

**Figure 2:**
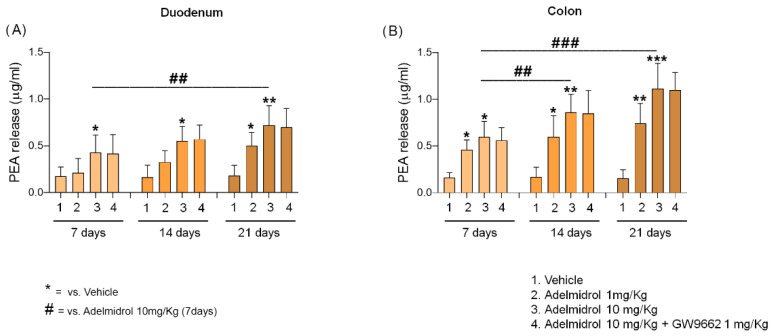
Effect of daily administration of Adelmidrol (1–10 mg/Kg) on PEA concentration in the duodenum (**A**) and colon (**B**) of treated mice, respectively, in the presence and absence of specific PPAR-γ inhibitor GW9662 (1 mg/Kg). PEA levels were measured at 7, 14, and 21 days. Results are expressed as mean ± SD of *n* = 4 experiments performed in triplicate. * *p* < 0.05, ** *p* < 0.01, and *** *p* < 0.001 respectively versus vehicle of the same timepoint. ## *p* < 0.01 and ### *p* < 0.001 respectively versus Adelmidrol (10 mg/Kg) at day 7.

Adelmidrol (1–10 mg/Kg) increased PEA concentration was administered daily to the duodenum and colon of treated mice. A statistically significant increase of PEA levels was observed in duodenal biopsies from the Adelmidrol 10 mg/kg group as early as at day 7 (+151% vs. vehicle, *p* < 0.05). The results obtained at day 14 in the same treatment group were similar (+246% vs. vehicle group, *p* < 0.05), demonstrating an upward trend in duodenal PEA concentration. At the end of the experiment (day 21) a statistically significant increase in PEA levels at the duodenal site was evident in the Adelmidrol 1 mg/Kg group (+208% vs. vehicle, *p* < 0.05). At the same timepoint, PEA duodenal levels continued to increase in the Adelmidrol 10 mg/Kg group (+330% vs. vehicle, *p* < 0.01, and +66% vs. Adelmidrol 10/mg/Kg at day 7, *p* < 0.01) (Figure 2A).

Observations at the colon site were almost comparable to those found at the duodenal level, with the difference that colonic levels of PEA were slightly higher. A significant increase of PEA was observed at day 7 in both Adelmidrol 1 mg/Kg (+176% vs. vehicle, *p* < 0.05) and Adelmidrol 10 mg/Kg (+258% vs. vehicle, *p* < 0.05) groups. The upward trend in PEA levels noticed at the duodenal site was confirmed in colonic biopsies at day 14, as demonstrated by the statistically significant increase of PEA concentration in both Adelmidrol 1 mg/Kg (+245 vs. vehicle, *p* < 0.05) and Adelmidrol 10 mg/Kg (+397 vs vehicle, *p* < 0.01, and +42% vs. Adelmidrol 10/mg/Kg at day 7, *p* < 0.05). At the last timepoint (day 21) the levels of PEA in the colon were in line with pervious results in both Adelmidrol 1 mg/Kg (+368% vs. vehicle, *p* < 0.01) and Adelmidrol 10 mg/Kg (+612% vs. vehicle, *p* < 0.001, and +87% vs. Adelmidrol 10/mg/Kg at day 7, *p* < 0.01) groups (Figure 2B). Interestingly, PPAR-γ inhibition did not affect the enhancing effect of Adelmidrol on PEA levels either at the duodenal or colonic sites (Figure 2A,B), demonstrating a substantial lack of correlation between Adelmidrol’s “entourage” effect and the agonism on this receptor.

### 2.2. Adelmidrol Increases NAPE-PLD and FAAH Proteins Expression and NAAA-Coding mRNA with a Dose/Time-Dependent Pattern in Colon and Duodenum

**Figure 3:**
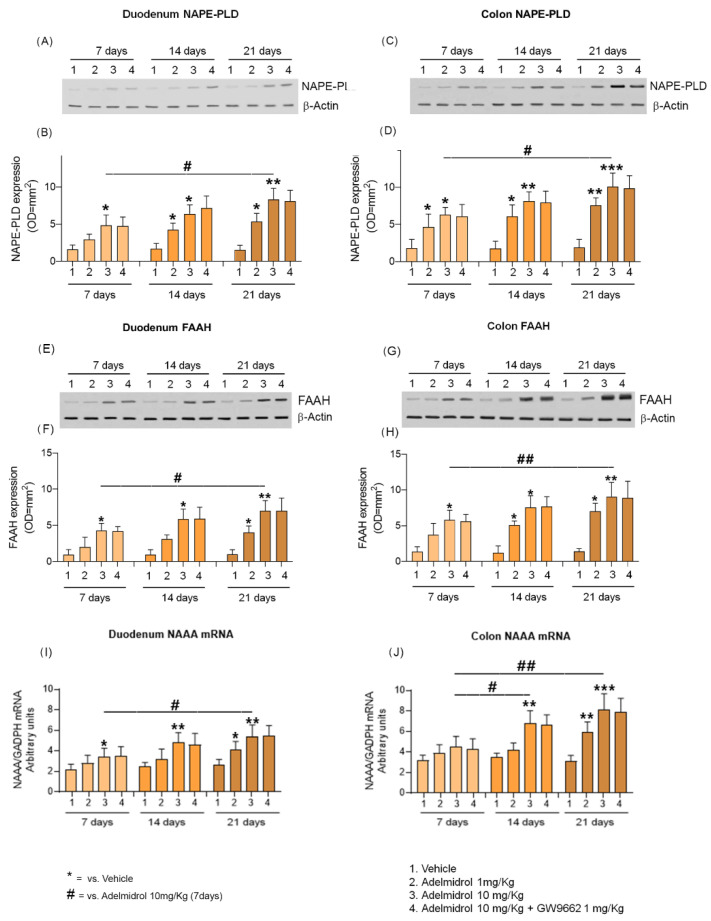
Effect of daily administration of Adelmidrol (1–10 mg/Kg) on NAPE-PLD and FAAH protein expression and NAAA-coding mRNA in the duodenum and colon of treated mice, respectively, in the presence and absence of specific PPAR-γ inhibitor GW9662 (1 mg/Kg). (**A**,**C**,**E**,**G**) Immunoreactive bands referred to NAPE-PLD and FAAH proteins expression measured at 7, 14, and 21 days in duodenum and colon, and (**B**,**D**,**F**,**H**) relative densitometric analysis of each protein (arbitrary units normalized on the expression of the housekeeping protein β-Actin). (**I**) The NAAA gene expression in duodenum measured at 7, 14, and 21 days. (**J**) The NAAA gene expression in colon measured at 7, 14, and 21 days. Results are expressed as mean ± SD of *n* = 4 experiments performed in triplicate. * *p* < 0.05, ** *p* < 0.01, and *** *p* < 0.001, respectively, versus vehicle of the same timepoint. # *p* < 0.05 and ## *p* < 0.01 versus Adelmidrol (10 mg/Kg) at day 7.

In addition to the increase of PEA levels, we observed a parallel enhancement in NAPE-PLD and FAAH protein expression. At day 7 duodenum samples from Adelmidrol 10 mg/Kg treated mice showed a significative increase in both NAPE-PLD and FAAH (+195% and +328% vs. vehicle; all *p* < 0.05, respectively; Figure 3A–F). The increase of NAPE-PLD protein expression continued until day 14 in both Adelmidrol 1 mg/Kg (+156% vs. vehicle, *p* < 0.05, Figure 3A,B) and Adelmidrol 10 mg/Kg (+280% vs vehicle, *p* < 0.05%, Figure 3A,B). Conversely, at day 14, FAAH significantly increased protein expression was only evident in the Adelmidrol 10 mg/Kg group (+525% vs vehicle, *p* < 0.05). At day 21 we observed a further increase in the upward trend of NAPE-PLD and FAAH expression in Adelmidrol 1 mg/Kg group (+265% vs. vehicle, *p* < 0.05, Figure 3A,B) (+307% vs. vehicle, *p* < 0.05, Figure 3E,F) as well as in the Adelmidrol 10 mg/Kg group (+473% vs. vehicle, *p* < 0.01, +73% vs. Adelmidrol 10 mg/Kg at day 7, *p* < 0.05, Figure 3A,B) (+625% vs. vehicle, *p* < 0.01, +70% vs. Adelmidrol 10 mg/Kg at day 7, *p* < 0.05, Figure 3E,F).

In parallel, RT-PCR analysis on intestinal samples revealed an increased transcription of NAAA-coding mRNA. After 14 days of treatment, a significant increase in duodenal NAAA mRNA transcription was observed in the Adelmidrol 10 mg/kg group (+101% vs. vehicle, *p* < 0.01), demonstrating an upward trend in duodenal NAAA expression. A week later (day 21) NAAA mRNA in duodenum was stable in the Adelmidrol 10 mg/Kg group (+111% vs. vehicle, *p* < 0.01, and +56% vs. Adelmidrol 10 mg/Kg at day 7, *p* < 0.05) (Figure 3A) and a statistically significant increase was also evident in the Adelmidrol 1 mg/Kg group (+59% vs. vehicle, *p* < 0.05) (Figure 3I).

As observed in the duodenum, the colon samples revealed a dose/time-dependent increase in NAPE-PLD and FAAH protein expression in response to Adelmidrol administration. At day 7 colon samples from both Adelmidrol 1 mg/Kg (+150% vs. vehicle, *p* < 0.05, Figure 3C,D) and Adelmidrol 10 mg/Kg (+233% vs. vehicle, *p* < 0.05, Figure 3C,D) groups showed a significant increase in NAPE-PLD expression. Similarly, FAAH protein expression was increased in the Adelmidrol 10 mg/Kg group at day 7 (+325% vs. vehicle, *p* < 0.05, Figure 3G,H). Even at day 14, a further increase in NAPE-PLD and FAAH proteins expression were observed in both Adelmidrol 1 mg/Kg (+235% vs. vehicle, *p* < 0.05, Figure 3C,D) (+269% vs. vehicle, *p* < 0.05, Figure 2G,H) and Adelmidrol 10 mg/Kg (+342% vs vehicle, *p* < 0.01%, Figure 3C,D) (+451% vs vehicle, *p* < 0.05%, Figure 3G,H) groups. At day 21 the upward trend of NAPE-PLD and FAAH protein expression was even more evident in both the Adelmidrol 1 mg/Kg group (+313% vs. vehicle, *p* < 0.01, Figure 3C,D) (+385% vs. vehicle, *p* < 0.05 Figure 3G,H) and the Adelmidrol 10 mg/Kg group (+514% vs. vehicle, *p* < 0.001, +70% vs. Adelmidrol 10/mg/Kg at day 7, *p* < 0.05, Figure 3C,D) (+625% vs. vehicle, *p* < 0.01, +70% vs. Adelmidrol 10 mg/Kg at day 7, *p* < 0.05, Figure 3G,H). As noted for PEA levels, the selective PPAR-γ antagonist GW9662 did not interfere with NAPE-PLD and FAAH up-regulation, at neither the colon nor the duodenum sites (Figure 3A–H).

A further analysis revealed that in the colon NAAA-coding mRNA was almost comparable to that observed in the duodenum, even if the average amount was slightly higher. A significant increase was observed at day 14 (+93% vs vehicle, *p* < 0.01, and +52% vs. Adelmidrol 1 mg/Kg at day 7, *p* < 0.05) in the Adelmidrol 10 mg/Kg group. At the last timepoint (day 21) the NAAA mRNA transcription in the colon was raised in both Adelmidrol 1 mg/Kg (+97% vs. vehicle, *p* < 0.01) and Adelmidrol 10 mg/Kg groups (+168% vs. vehicle, *p* < 0.001, and +81% vs. Adelmidrol 10 mg/Kg at day 7, *p* < 0.01) (Figure 3B). PPAR-γ inhibition did not show any effect on the increase of the NAAA-coding mRNA transcription induced by Adelmidrol administration (Figure 3J).

### 2.3. Adelmidrol Increases PEA Release, Reduces Inflammatory Markers and Cells Viability in Caco-2 Cell Line

To further explain the effects of Adelmidrol-induced PEA increases, additional experiments were performed in Caco-2 cells mimicking an inflammatory condition.

**Figure 4:**
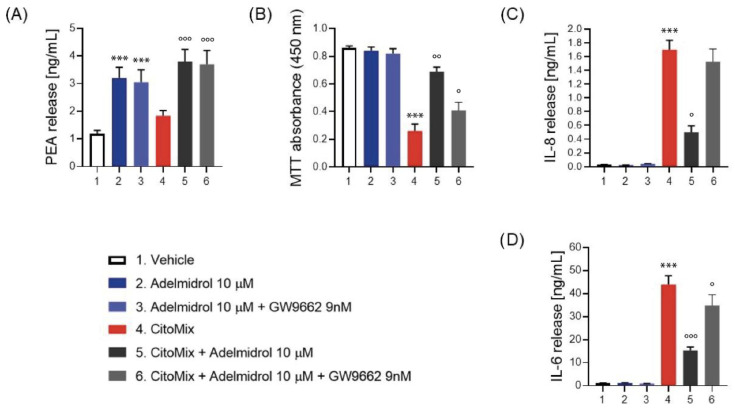
Effect of 24 h incubation of Adelmidrol (10 μM) on Caco-2 cells in the presence and absence of CytoMix (IFN-γ, 1000 U/mL; TNF-α, 10 ng/mL; and IL-1β, 1 ng/mL) and in the presence and absence of specific PPAR-γ inhibitor GW9662 (9 nM). (**A**) PEA levels in Caco-2 cells after 24 h incubation. (**B**) MTT absorbance in Caco-2 cells supernatant after 24 h incubation. (**C**) IL-8 and (**D**) IL-6 levels in Caco-2 cells supernatant after 24 h incubation. Results are expressed as mean ± SD of *n* = 4 experiments performed in triplicate. *** *p* < 0.001 versus vehicle. ° *p* < 0.05, °° *p* < 0.01, and °°° *p* < 0.001 versus CytoMix group.

After 24 h of incubation of Adelmidrol, PEA production in Caco-2 cells were increased in resting cells in both the presence and absence of GW9662 (group 2, +68% vs. group 1, *p* < 0.001) (group 3, +60% vs. group 1, *p* < 0.001). Similar outcomes were observable in Caco-2 cells incubated with CytoMix (group 5, +107% vs. group 3, *p* < 0.001) (group 6, +94% vs. group 3, *p* < 0.001). Both in resting and in pro-inflammatory conditions the inhibition of PPAR-γ did not influence the increase in PEA (Figure 4A).

Moreover, CytoMix incubation triggered a massive release of pro-inflammatory cytokines, such as IL-8 and IL-6. On the other hand, Adelmidrol incubation reduced such inflammatory markers (IL-8, −70% vs. group 4, *p* < 0.001) (IL-6, −65% vs. group 4, *p* < 0.001). Also in this case, the presence of GW9662 was not able to completely abolish the anti-inflammatory effect of Adelmdidrol. Indeed, levels of IL-6 in group 6 were lower when compared to those detected in group 4 (−25% vs. group 4, *p* < 0.05).

As a further confirmation of the safety of Adelmidrol, after 24 h incubation we did not observe any reduction of cell viability. Conversely, Cytomix incubation significantly reduced cell viability (−70% vs. group 1, *p* < 0.001), but these effects were almost completely reversed by the presence of Adelmidrol 10 μM (+165% vs. group 4, *p* < 0.01). Interestingly, the incubation with GW9662 did not completely abolish Adelmidrol’s protective effect on enterocytes (+58% vs. group 4, *p* < 0.05).

## 3. Discussion

The present study demonstrated that Adelmidrol oral administration (1 and 10 mg/kg) increased the endogenous concentration of PEA in mouse duodenal and colonic biopsies, probably due to an “entourage” effect on metabolic pathways, with PPAR-γ not being involved. Our data are in line with what was previously demonstrated in vitro regarding the ability of Adelmidrol to increase PEA levels [25]. More generally, our data agree with the so-called “entourage hypothesis”, i.e., the enhancing effect exerted by phytocannabinoids and ALIAmides on the levels and/or receptor affinities of endogenous protective compounds, such as anandamide (AEA), oleoylethanolamide (OEA), and 2-arachidonoyl glycerol (2-AG) and PEA [13,26,27,28]^.^ Specifically, the ability of PEA to both increase the endogenous levels and the actions at TRPV1 channels of AEA and arachidonoylglycerol (2-AG) were explained through its “entourage effect” [13,28,29,30]. 

Moreover, we demonstrated that oral administration of Adelmidrol can enhance the tissue levels of PEA at either the colonic or duodenal sites independently of the interaction with PPAR-γ. In this regard, a detailed study by Cordaro et al. [22] demonstrated the PPAR-γ-dependent anti-inflammatory action exerted by Adelmidrol in a model of DNBS-induced colitis in mice, suggesting that the effect of such a molecule mostly depends on the activation of the above-mentioned receptor at the intestinal site. Conversely, in our experimental conditions Adelmidrol resulted in increased intestinal PEA levels without interacting with PPAR-γ, thus configuring Adelmidrol as a sort of “chemical patch” able to trigger duodenal and colonic PEA production, regardless of a specific pharmacological target. The increased levels of PEA in the intestine are the main findings that give confirmation that Adelmidrol exerts an entourage effect at this site.

Since PEA hydrolysis to palmitic acid and ethanolamine is mostly due to catabolic enzymes NAAA and FAAH [31,32], we investigated their possible changes, in terms of protein expression, as a consequence of homeostatic reactions aimed at balancing PEA increase. Surprisingly, we evidenced a time/dose-dependent increase in protein expression of FAAH as well as an augmented transcription of NAAA-coding mRNA in response to Adelmidrol administration. A previously reported in vitro enzymatic assay, aimed at understanding the potential inhibitory effect of Adelmidrol on NAAA and FAAH, demonstrated the absence of Adelmidrol-related inhibitory proprieties in these enzymes [25]. Such results suggest an endogenous negative control exerted by the main enzymes catalysing PEA catabolism aimed at balancing the “entourage effect” of Adelmidrol. On the other hand, the lack of efficacy of Adelmidrol in interfering with NAAA and FAAH hydrolytic activity shows that enhanced PEA concentration, detected in the present and in previous studies, does not depend on an inhibition of these two enzymes. A different hypothesis could be that such an increase in catabolic enzyme levels depend on the fact that Adelmidrol increases the endogenous release of other substrates of FAAH. That could explain why the rise of FAAH expression is not accompanied with a decrease in endogenous PEA release.

In order to explain the reason for PEA increase following Adelmidrol oral administration, we evaluated the expression of NAPE-PLD, the main biosynthetic enzyme of NAEs [33,34,35]. As expected, we noticed a significant up-regulation of NAPE-PLD in both the duodenum and colon from day 7, which may explain the augmented PEA concentration in these sites. Anyway, the exact mechanism by which Adelmidrol induces NAPE-PLD up-regulation is unclear, and further studies will be necessary to explain this data. In any case, a probable explanation for our findings may lie in an indirect consequence due to the “entourage effect” exerted by Adelmidrol that may increase NAPE-PLD expression. After all, if Adelmidrol directly interacted with NAPE-PLD, enhancing its activity, we should have, *bona fide*, observed a physiological downregulation, due to negative feedback aimed at reducing PEA biosynthesis. In contrast with our findings, Petrosino et al. reported downregulation of NAPE-PLD exerted by Adelmidrol in keratinocytes, and their evidence was rightly explained by citing that NAPE-PLD is not the major PEA biosynthetic enzyme in that cell subtype [25]. This additional data suggests that the indirect mechanism by which Adelmidrol increases PEA concentration may be more complex, involving different pathways.

**Figure 5:**
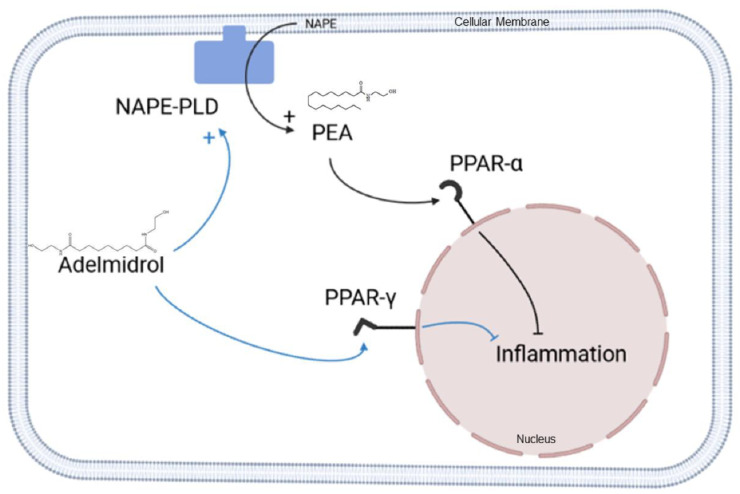
Adelmidrol’s main anti-inflammatory mechanism of action (blue) exerted via PPAR-γ agonism and up-regulating NAPE-PLD enzyme expression. Side-on adjuvant mechanism (Black) exerted by the increased PEA production via PPAR-α agonism. NAPE = N-acyl phosphatidylethanolamine; PLD = Phospholipase D; PEA = Palmitoylethanolamide; PPAR = Peroxisome proliferator-activated receptor.

In parallel, present in vitro results, obtained on Caco-2 cells, confirm that Adelmidrol can up-regulate PEA levels in intestinal epithelium even in inflammatory conditions. This was predictable, since PEA is naturally released at the intestinal site in response to damage and inflammatory conditions. Moreover, these results give us further confirmation of the PPAR-γ-independent mechanism by which Adelmidrol increases the endogenous levels of PEA. Indeed, the presence of PPAR-γ inhibitor did not completely revert the protecting effect of Adelmidrol on cell viability and did not completely abolish the reduction of IL-6 exerted by this treatment. It is possible that such results depend upon the protective effect exerted by the increased levels of PEA triggered, by Adelmidrol (see Figure 5). 

Considering the well-established analgesic, immunomodulatory, and anti-inflammatory actions exerted by PEA on intestinal disorders, and promising perspectives opened by Adelmidrol pre-clinical data in IBD management [18,22], future studies may be conducted to evaluate the possible potential of this molecule. The marked “entourage” effect exerted by Adelmidrol, combined with its anti-inflammatory PPAR-γ-dependent effect, may be promising in clinical and subclinical conditions, like irritable bowel syndrome (IBS) and unbalanced diets, in which lower PEA levels has been reported, either in experimental and clinical settings [36,37,38,39,40]. Furthermore, this intriguing perspective may open up testing of Adelmidrol in dyspeptic models, in which the protective role of PEA has been established. Also, Adelmidrol, as well as PEA and the others ALIAmides, is considered to be a safe compound in both humans and animals and is widely used in clinical and veterinary practices [19,23,41]. It is therefore likely that transnationality of such results in humans will be probable. Future studies will be needed to better understand the contribution exerted in vivo by increased PEA production in resolving colitis, as well as to gain a deeper characterization of Adelmidrol’s effect on immune cells (such as macrophages and lymphocytes) heavily involved in these conditions.

In conclusion, our study demonstrated, for the first time, that oral administration of Adelmidrol enhanced the endogenous levels of PEA at either the duodenal or colonic site, paving the way for a better understanding of its mechanism of action, and highlighting the role of increased PEA production.

## 4. Materials and Methods

### 4.1. Animals and Experimental Design

**Figure 6:**
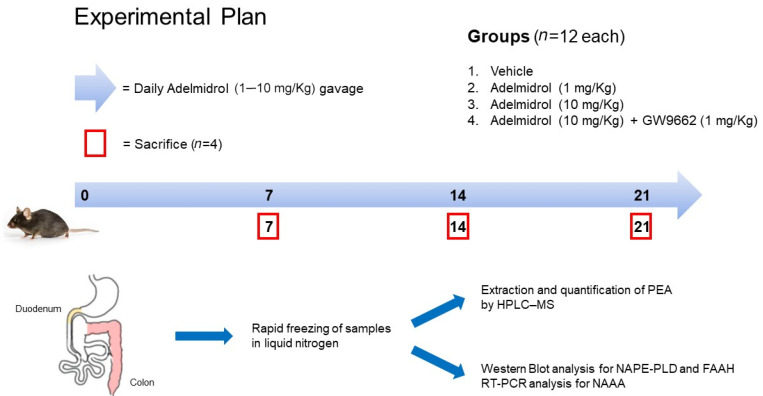
Experimental plan.

All experiments involving animals were carried out according to Sapienza University’s Ethics Committee, approval code 890/2021-PR, approved on 17 November 2021. Animal care followed the IASP and European Community (EC L358/1 18/12/86) guidelines on the use and protection of animals in experimental research. Eight-week-old male C57BL/6 mice were used for the experiments (Charles River, Lecco, Italy). All mice were maintained on a 12-h light/dark cycle in a temperature-controlled environment with access to food and water ad libitum. Mice were randomly divided in four groups (12 mice/group): (1) vehicle group, mice treated with daily gavage of 150 µL of phosphate buffer solution (PBS) 1X; (2) Adelmidrol 1 mg/Kg, mice treated with daily gavage of 150 µL of Adelmidrol 1 mg/Kg in PBS 1X; (3) Adelmidrol 10 mg/Kg, mice treated with daily gavage of 150 µL of Adelmidrol 10 mg/Kg in PBS 1X; (4) Adelmidrol 10 mg/Kg + GW9662 1 mg/Kg, mice treated with daily gavage of 150 µL of Adelmidrol 10 mg/Kg in PBS 1X and weekly intraperitoneal puncture (IP) of GW9662 1 mg/Kg in sterile saline solution. The experimental protocol lasted 21 consecutive days and *n* = 4 animals were sacrificed at day 7, 14, and 21. Samples of duodenum and colon were collected, rapidly frozen in liquid nitrogen, and stored at −80°C until analysis (See Figure 6).

### 4.2. Extraction and Quantification of In Vivo and In Vitro Produced PEA by HPLC–MS Method

Extraction and analysis of PEA levels were performed according to Gachet et al. [42], with slight modifications. Mice colonic and duodenal tissues and Caco-2 pellets were firstly lysed, using a lysis buffer, and then evaporated under a nitrogen stream. Residues were suspended in extraction solution, ultracentrifuged (14,000 rpm, 4 °C, 5 min), and the supernatant injected for mass spectrometry analysis. Analyses were run on a Jasco Extrema LC-4000 system (Jasco Inc., Easton, MD, USA) coupled to an Advion Expression mass spectrometer (Advion Inc., Ithaca, NY, USA) equipped with an electrospray (ESI) source. Mass spectra were recorded in the positive SIM mode. The capillary voltage was set at +180 V, the spray voltage was at 3 kV, the source voltage offset was at +20 V, and the capillary temperature was set at 250 °C. The chromatographic separation was performed on analytical column Kinetex C18 (150 × 4.6 mm, id. 3 μm, 100 Å) and a security guard column, both supplied by Phenomenex (Torrance, CA, USA). The analyses were performed at a flow rate of 0.3 mL/min, with solvent A (water containing 2 mM ammonium acetate) and solvent B (methanol containing 2 mM ammonium acetate and 0.1% formic acid). Elution was performed according to the following linear gradient: 15% B for 0.5 min, 15–70% B from 0.5 to 2.5 min, 7–99% B from 2.5 to 4.0 min, and held at 99% B from 4.0 to 8.0 min. From 8 to 11.50 min, the column was equilibrated to 15% B and conditioned from 11.5 to 15.0 at 15% B. The injection volume was 10 μL, and the column temperature was fixed at 40 °C. For quantitative analysis, standard curves of PEA (Sigma-Aldrich, Milan, Italy) were prepared over a concentration range of 0.0001-10 ppm with six different concentration levels, and duplicate injections were prepared at each level. All data were collected and processed using JASCO Chrom NAV (version 2.02.04) and Advion Data Express (4.0.13.8).

### 4.3. Western Blot Analysis

Proteins were extracted from colonic and duodenal tissue and processed by Western blot analysis. Tissue samples were homogenized in ice-cold hypotonic lysis buffer [10 mM 4-(2-hydroxyethyl)-1-piperazineethanesulfonic acid (HEPES), 1.5 mM MgCl_2_, 10 mM KCl, 0.5 mM phenylmethylsulphonylfluoride, 1.5 μg/mL soybean trypsin inhibitor, 7 mg/mL pepstatin A, 5 mg/mL leupeptin, 0.1 mM benzamidine, and 0.5 mM dithiothreitol (DTT)]. Tissue-deriving protein extracts were mixed with a nonreducing gel loading buffer [50 mM Tris (hydroxymethyl)aminomethane (Tris), 10% sodium dodecyl sulfate (SDS), 10% glycerol, 2 mg/mL bromophenol] at a 1:1 ratio, and then boiled for 3 min followed by centrifugation at 10,000× g for 10 min. The protein concentration was determined using Bradford assay and equivalent amounts (50 μg) of each homogenate underwent electrophoresis through a polyacrylamide mini-gel. After the transfer, the membranes were incubated with 10% nonfat dry milk in PBS 1X overnight at 4°C and then exposed, depending on the experiments, with rabbit polyclonal anti-NAPE-PLD (Abcam, Cambridge, United Kingdom) (1:200 *v/v*) and mouse monoclonal anti–FAAH (MERK KGaA, Darmstadt, Germany) (3 μg/mL *w/v*), according to standard experimental protocols. Membranes were then incubated with the specific secondary antibodies conjugated to HRP (Dako, Milan, Italy). Immune complexes were exposed to enhanced chemiluminescence detection reagents, and the blots were analyzed by scanning densitometry (VersadocMP4000; Bio-Rad, Segrate, Italy). Results were expressed as optical density (OD; arbitrary units/mm^2^) and normalized against the expression of the housekeeping protein β-actin.

### 4.4. RT-PCR Analysis

RNA was isolated using TRIzol from duodenum and colon samples of treated mice. CDNAs were reverse transcribed from total RNA (0.5 μg) by using PrimeScript RT reagent kit, according to the manufacturer’s instructions (Takara Bio). Real-time PCR analyses were then performed with ABI Prism 7000 Sequence Detection System (Life Technologies) using SYBR Premix Ex TaqII to evaluate the hydrolytic enzyme NAAA: forward 5′-AAGGCTGGTGGTGGGAGAA-3′ and reverse 5′-TCAGCAATGAGGGGAGTCTTG-3′. The NAAA gene expression was normalized to endogenous housekeeping gene glyceraldehyde-3-phosphate dehydrogenase (GAPDH): forward 5′-AACTCCCACTCTTCCACCTTCGATG-3′ and reverse 5′-CCTGTTGCTGTAGCCGTATTCATTG-3′. The RT-PCR condition was as follows: denaturation at 95 °C for 5 s and annealing/extension at 60 °C for 31 s (40 cycles) [43].

### 4.5. Cells Culture

Caco-2 cells were cultured in 6-well plates in Dulbecco’s Modified Eagle Medium (DMEM) containing 10% foetal bovine serum (FBS), 1% penicillin–streptomycin, 2 mM L-glutamate, and 1% non-essential amino acids. A total of 1 × 10^6^ cells/well were plated and incubated for 24 h in a humidified atmosphere of 5% CO_2_ at a temperature of 37 °C in Midi 40 CO_2_ incubator (Thermo Fisher Scientific Inc., Waltham, MA, USA). Upon reaching confluence, cells were washed three times with phosphate-buffered saline (PBS), detached with trypsin/ethylenediamine tetra acetic acid (EDTA), and, dependent on the experiment, plated in 6-well plates, 96-well plates, or 10 cm diameter Petri dishes, and allowed to adhere for a further 24 h. Cells were divided in *n* = 6 groups: (1) Vehicle; (2) Adelmidrol 10 μM; (3) Adelmidrol 10 μM + GW9662 9 nM; (4) CytoMix (IFN-γ, 1000 U/mL; TNF-α, 10 ng/mL; and IL-1β, 1 ng/mL, R and D Systems, Inc., Minneapolis); (5) CytoMix + Adelmidrol 10 μM; (6) CytoMix + Adelmidrol 10 μM + GW9662 9 nM.

### 4.6. Cytotoxicity Assay

Proliferation and survival of the Caco-2 cells were assayed using the MTT reagent. The cells in DMEM (5 × 10^4^ cells/well) were plated in 96-well plates and allowed to adhere for 24 h. The medium was then replaced, and the cells underwent treatments as described above. After 24 h, 25 μL of MTT stock solution in DMEM (5 mg/mL) was added to the cells and then incubated for 3 h at 37 °C. Subsequently, the cells were lysed, and the dark blue crystals were solubilized using 100 μL solution, containing 50% N, N-dimethylformamide, and 20% (*w/v*) SDS (pH = 4.5). The OD of each well was determined at 450 nm using a microplate spectrophotometer (PerkinElmer, Inc; Waltham, MA, USA). 

### 4.7. ELISA

Enzyme-linked immunosorbent assay (ELISA) for human IL-8 and IL-6 (KeyGEN, Nanjing, China) were carried out on Caco-2 cell supernatant after treatments at 24 h, according to the manufacturer’s protocol. Absorbance was measured on a microtiter plate reader. IL-8 and IL-6 levels were thus determined using the standard curves method.

### 4.8. Statistical Analysis

Data were analyzed using GraphPad Prism 8 (GraphPad Software, San Diego, CA, USA) by one-way analysis of variance (ANOVA) with a Bonferroni post-test or unpaired *t*-test, as appropriate, with *p* < 0.05 considered statistically significant. Results are shown as means ± standard deviation (SD) of *n* = 4 experiments performed in triplicate for each experimental group at each time point.

## Data Availability

The data presented in this study are available on request from the corresponding author. The data are not publicly available due to the policy of our research group, we will share data unreservedly on on-line data sharing platform upon request.
